# Hepatitis C virus viremic rate in the Middle East and North Africa: Systematic synthesis, meta-analyses, and meta-regressions

**DOI:** 10.1371/journal.pone.0187177

**Published:** 2017-10-31

**Authors:** Manale Harfouche, Hiam Chemaitelly, Silva P. Kouyoumjian, Sarwat Mahmud, Karima Chaabna, Zaina Al-Kanaani, Laith J. Abu-Raddad

**Affiliations:** 1 Infectious Disease Epidemiology Group, Weill Cornell Medicine-Qatar, Cornell University, Qatar Foundation - Education City, Doha, Qatar; 2 Department of Healthcare Policy & Research, Weill Cornell Medicine, Cornell University, New York, United States of America; University of North Carolina at Chapel Hill School of Medicine, UNITED STATES

## Abstract

**Objectives:**

To estimate hepatitis C virus (HCV) viremic rate, defined as the proportion of HCV chronically infected individuals out of all ever infected individuals, in the Middle East and North Africa (MENA).

**Methods:**

Sources of data were systematically-gathered and standardized databases of the MENA HCV Epidemiology Synthesis Project. Meta-analyses were conducted using DerSimonian-Laird random-effects models to determine pooled HCV viremic rate by risk population or subpopulation, country/subregion, sex, and study sampling method. Random-effects meta-regressions were conducted to identify predictors of higher viremic rate.

**Results:**

Analyses were conducted on 178 measures for HCV viremic rate among 19,593 HCV antibody positive individuals. In the MENA region, the overall pooled mean viremic rate was 67.6% (95% CI: 64.9–70.3%). Across risk populations, the pooled mean rate ranged between 57.4% (95% CI: 49.4–65.2%) in people who inject drugs, and 75.5% (95% CI: 61.0–87.6%) in populations with liver-related conditions. Across countries/subregions, the pooled mean rate ranged between 62.1% (95% CI: 50.0–72.7%) and 70.4% (95% CI: 65.5–75.1%). Similar pooled estimates were further observed by risk subpopulation, sex, and sampling method. None of the hypothesized population-level predictors of higher viremic rate were statistically significant.

**Conclusions:**

Two-thirds of HCV antibody positive individuals in MENA are chronically infected. Though there is extensive variation in study-specific measures of HCV viremic rate, pooled mean estimates are similar regardless of risk population or subpopulation, country/subregion, HCV antibody prevalence in the background population, or sex. HCV viremic rate is a useful indicator to track the progress in (and coverage of) HCV treatment programs towards the set target of HCV elimination by 2030.

## Introduction

Viral hepatitis is ranked as the 7^th^ leading cause of mortality worldwide [1], with nearly half of this mortality attributed to hepatitis C virus (HCV) [1]. Despite its global burden, the Middle East and North Africa (MENA) remains the most affected region [2, 3]. With the advent of direct-acting antivirals (DAAs) to treat and cure HCV infection [4], a global target was set to eliminate HCV infection by 2030 [5, 6].

A key feature of HCV natural history is that not all infected persons develop chronic infection [7–9]. While infected persons pass through a stage of acute infection for few months, and develop antibodies against HCV infection, a proportion of them spontaneously clear the infection and becomes HCV antibody (Ab) positive but HCV ribonucleic acid (RNA) negative [7–9]. The remainder of infected persons become chronic carriers of the infection and persist as HCV Ab positive and RNA positive [7–9]. For a given population, the proportion of chronically infected individuals (HCV Ab positive and RNA positive), out of all ever infected individuals (HCV Ab positive regardless of RNA status), defines the *HCV viremic rate* for this population [10].

Assessing and understanding the HCV viremic rate is critical for biological, epidemiological, and public health consequences. The HCV viremic rate provides a measure of HCV spontaneous clearance rate and its determinants, and how this rate may vary by population [11]. The HCV viremic rate furnishes also a direct measure of the likelihood that a member of a specific population is chronically infected, as well as an indirect measure of the risk of HCV reinfection in this population [11]. It is further essential for estimations of the number of HCV chronic carriers in different populations and countries, and consequences for resource allocation and development of screening and treatment programs. The HCV viremic rate will also play an increasingly important role in assessing and monitoring the progress in (and coverage of) HCV treatment programs in different populations, as we forge ahead towards HCV elimination by 2030.

The HCV viremic rate has been assessed through numerous studies in different populations globally, but its measures show extensive variability across studies [12–21]. The HCV clearance rate, which is strongly linked to HCV viremic rate [11], has been also assessed in multiple prospective cohort studies [7, 22–24], but its measures also show wide variation across studies [11]. To our knowledge, no study have yet been conducted to provide an overall pooled estimate and subgroup pooled estimates for the HCV viremic rate that factor the wide diversity of studies for this measure. No study has also investigated the sources of heterogeneity in available HCV viremic rate measures.

Against this background, we aimed in the present study to provide pooled estimates for the HCV viremic rate, overall and for different risk populations and different countries of the MENA region. We also aimed to investigate the sources of heterogeneity in available measures in MENA. These quantitative assessments were based on a comprehensive and standardized database of systematically gathered HCV viremic rate data.

This study was conducted as part of the MENA HCV Epidemiology Synthesis Project, an ongoing effort to characterize HCV epidemiology and inform public health research, resource allocation, policy, and programing priorities in MENA [11–21, 25, 26].

## Methodology

### Data sources

We retrieved studies reporting HCV RNA prevalence measures *strictly* among HCV Ab positive individuals from the MENA HCV Epidemiology Synthesis Project databases. These databases consist of 2,543 studies reporting HCV Ab prevalence among 52,598,736 participants, 47 studies reporting HCV Ab incidence among 29,600 participants, and 338 studies reporting HCV genotypes among 82,257 participants. The retrieved HCV RNA prevalence measures were nearly always extracted from studies whose main outcome measure was HCV Ab prevalence in some specific population. HCV RNA prevalence was a secondary outcome of these studies.

The HCV Synthesis Project databases were compiled through systematic reviews of the literature [12–17, 19–21] that were informed by the Cochrane Collaboration handbook [27], and reported as per the Preferred Reporting Items for Systematic Reviews and Meta-analyses (PRISMA) guidelines [28]. The literature searches were conducted using broad search criteria with no language or year restrictions (S1 and S2 Boxes), and were based on international databases (PubMed and Embase), regional databases, national databases, and the MENA HIV/AIDS Epidemiology Synthesis Project database [29, 30]. Separate searches were also conducted for the non-indexed literature consisting of public health reports and routine data reporting. The flowcharts summarizing the searches can be found in the previous publications [12–17, 19–21]. The PRISMA checklist for the present study can be found in S1 Fig.

The definition of MENA for this project consisted of 24 countries including: Afghanistan, Algeria, Bahrain, Djibouti, Egypt, Iran, Iraq, Jordan, Kuwait, Lebanon, Libya, Mauritania, Morocco, Oman, Pakistan, Palestine, Qatar, Saudi Arabia, Somalia, Sudan, Syria, Tunisia, the United Arab Emirates (UAE), and Yemen.

### Study selection and classification

All studies reporting a measure of HCV viremic rate were included, provided the sample size was ≥10. The HCV viremic rate was defined as the proportion of HCV Ab positive and RNA positive individuals out of all ever infected (i.e. HCV Ab positive) individuals in the sample. The overall sample size was replaced by stratified measures whenever this was possible while maintaining a subsample size ≥10. The viremic rate measures were classified based on the perceived risk of HCV exposure, as informed by existing classifications [2, 12–17, 19–21], as follows:

1. General populations: populations at a low risk of being exposed to HCV infection such as blood donors, healthy adults, healthy children, and pregnant women, among others. Those referred to in included studies as “general populations” were labeled as *other general populations* to avoid confusion with the name of this category.
2. Populations at intermediate risk: populations at an intermediate risk of being exposed to HCV infection such as health care workers, diabetics, and prisoners, among others).
3. Populations at high risk of healthcare-related exposures: populations at a high risk of being exposed to HCV infection due to a medical condition that requires frequent injections or blood transfusions such as hemodialysis, thalassemia, and hemophilia patients, among others.
4. People who inject drugs (PWID) who are at a high risk of being exposed to HCV infection due to sharing of needles or syringes.
5. Populations with liver-related conditions: populations suffering from liver-related medical conditions that could be linked (or attributed) to HCV infection, such as viral hepatitis, hepatocellular carcinoma, and liver cirrhosis patients, among others.
6. Special clinical populations: populations with an undetermined risk of HCV exposure such as patients with malignancies, rheumatology disorders, and autoimmune diseases, among others.

### Quantitative analysis

#### Meta-analyses

We conducted meta-analyses to estimate the pooled mean HCV viremic rate for the different risk populations. The methods were adapted from earlier meta-analyses [12–21, 26]. We used DerSimonian-Laird random-effects models with inverse variance weighting whenever we had ≥3 measures to be pooled [31]. The Freeman-Tukey type arcsine square-root transformation was used to stabilize the variance of the proportion measures [32]. Heterogeneity in effect size between studies was assessed using the Cochran’s Q test; a p-value <0.1 was considered significant [33, 34]. The I^2^ was used to assess the between-study variation associated with differences in effect size [33]. The prediction interval was calculated to identify the range where the true effect around the mean falls [33, 35].

Since we did not have sufficient number of studies to do a separate meta-analysis for each individual MENA country, we conducted meta-analyses by country or relevant subregional grouping (Afghanistan and Pakistan, Egypt, Fertile Crescent, Gulf, Iran, and Maghreb). The Fertile Crescent included Iraq, Jordan, Lebanon, Palestine, and Syria. The Gulf included Kuwait, Oman, and Saudi Arabia. The Maghreb included Algeria, Libya, Morocco, and Tunisia. This country/subregion classification included all MENA countries for which data on HCV viremic rate were available.

We further conducted meta-analyses for specific subpopulations among the general population (blood donors, children, pregnant women/antenatal care attendees, and other general populations), and specific subpopulations among the populations at high risk of healthcare-related exposures (hemophilia patients, hemodialysis patients, and thalassemia patients). We also conducted meta-analyses by sex (women only, men only, and mixed-sex), and by sampling method of the original study (convenience sampling, national population-based and probability-based sampling, and other probability-based sampling).

The meta-analyses were conducted using R studio version 3.3.2 [36] using the package meta [37].

#### Meta-regressions and sources of heterogeneity

We conducted univariable and multivariable random-effects meta-regressions to identify the predictors of higher HCV viremic rate and sources of between-study heterogeneity. The following independent variables were specified *a priori* because of epidemiological relevance: risk population, country or subregion, sex, age, HCV Ab prevalence of the sampled population, year of data collection, sample size, and sampling method. Variables with a p-value <0.1 in the univariable analyses were eligible for inclusion in the final multivariable model. Variables with a p-value <0.05 in the univariable models or the final multivariable model were considered statistically significant.

Risk population, country/subregion, and sex variables were categorized as described in the above sections. Age was categorized as children and adults. HCV Ab prevalence variable was coded as a categorical variable with four prevalence ranges: 1–10%, 10–30%, 30–50%, and >50%. The year of data collection variable was coded as a categorical variable with two date ranges: before 2000 and 2000 and thereafter. The sample size variable was categorized as ≥50 and <50. The sampling method variable was categorized as probability-based sampling and non-probability-based sampling.

For the year of data collection variable, we imputed the missing observations using the median of the results of the subtraction of the year of data collection from the year of publication. A sensitivity analysis using the imputed and the non-imputed observations revealed no impact on the statistical significance of the variable.

The meta-regressions were conducted using Stata/SE version 13 [38] using the package metareg [39].

## Results

### Scope of evidence

We identified 178 measures for HCV viremic rate among 19,593 HCV Ab positive individuals (Table 1). These measures included 81 in general populations, 20 in populations at intermediate risk, 51 in populations at high risk of healthcare-related exposures, five in PWID, eight in populations with liver-related conditions, and 12 in special clinical populations. One study was considered as “mixed” since the sample included a mix of general populations and an intermediate risk population [40].

**Table 1:** Studies reporting hepatitis C virus (HCV) viremic rate stratified by risk population across countries of the Middle East and North Africa.

Country	First author, year of publication	Years of data collection	Population description	Number of HCV Ab positive individuals tested for RNA	HCV viremic rate (%)
**General populations (n = 81)**					
**Egypt**	Abdel-Aziz, 2000 [46]	1997	Participants in a population-based survey	973	65.5
	AbdulQawi, 2011 [47]	2003–08	Pregnant women/ANC attendees	105	79.0
	Agha, 1998 [48]	1996–97	Pregnant women/ANC attendees	67	26.9
	Aguilar, 2008 [49]	-	General population	40	67.5
	Aguilar, 2008 [49]	-	General population	33	72.0
	Arafa, 2005 [50]	2002–03	Participants in a population-based survey	456	59.9
	Barakat, 2011 [51]	2005	Children	29	75.9
	Cowgill, 2004 [52]	1999–03	General population	80	65.0
	Darwish, 1995 [53]	-	Participants in a population-based survey	25	76.0
	Derbala, 2014 [54]	2008–10	General population	315	100
	El-Kamary, 2015 [55]	2012–13	Pregnant women/ANC attendees	52	58.0
	El-Karaksy, 2010 [56]	2006–07	Children	15	33.3
	El-Sadawy, 2004 [57]	-	Participants in a population-based survey	367	29.7
	El-Sherbini, 2003 [58]	1994	Children	17	41.0
	El-Zanaty, 2008 [41]	2008	15–19 years age group females in a national-based survey	178	67.8
	El-Zanaty, 2008 [41]	2008	20–24 years age group females in a national-based survey	153	66.9
	El-Zanaty, 2008 [41]	2008	25–29 years age group females in a national-based survey	94	62.9
	El-Zanaty, 2008 [41]	2008	30–34 years age group females in a national-based survey	134	70.2
	El-Zanaty, 2008 [41]	2008	35–39 years age group females in a national-based survey	129	69.3
	El-Zanaty, 2008 [41]	2008	40–44 years age group females in a national-based survey	122	65.2
	El-Zanaty, 2008 [41]	2008	45–49 years age group females in a national-based survey	109	60.0
	El-Zanaty, 2008 [41]	2008	50–54 years age group females in a national-based survey	85	70.4
	El-Zanaty, 2008 [41]	2008	55–59 years age group females in a national-based survey	107	68.8
	El-Zanaty, 2008 [41]	2008	10–14 years age group males in a national-based survey	68	76.7
	El-Zanaty, 2008 [41]	2008	20–24 years age group males in a national-based survey	65	62.6
	El-Zanaty, 2008 [41]	2008	25–29 years age group males in a national-based survey	62	73.8
	El-Zanaty, 2008 [41]	2008	30–34 years age group males in a national-based survey	59	53.6
	El-Zanaty, 2008 [41]	2008	35–39 years age group males in a national-based survey	54	66.2
	El-Zanaty, 2008 [41]	2008	40–44 years age group males in a national-based survey	50	61.1
	El-Zanaty, 2008 [41]	2008	45–49 years age group males in a national-based survey	42	65.0
	El-Zanaty, 2008 [41]	2008	50–54 years age group males in a national-based survey	32	74.6
	El-Zanaty, 2008 [41]	2008	55–59 years age group males in a national-based survey	28	71.1
	MoHP, 2015 [42]	2015	1–14 years age group females in a national-based survey	183	75.0
	MoHP, 2015 [42]	2015	15–19 years age group females in a national-based survey	164	66.5
	MoHP, 2015 [42]	2015	20–24 years age group females in a national-based survey	154	66.4
	MoHP, 2015 [42]	2015	25–29 years age group females in a national-based survey	61	81.1
	MoHP, 2015 [42]	2015	30–34 years age group females in a national-based survey	142	63.7
	MoHP, 2015 [42]	2015	35–39 years age group females in a national-based survey	106	69.5
	MoHP, 2015 [42]	2015	40–44 years age group females in a national-based survey	102	69.8
	MoHP, 2015 [42]	2015	45–49 years age group females in a national-based survey	85	75.1
	MoHP, 2015 [42]	2015	50–54 years age group females in a national-based survey	60	73.9
	MoHP, 2015 [42]	2015	55–59 years age group females in a national-based survey	69	73.4
	MoHP, 2015 [42]	2015	1–9 years age group males in a national-based survey	53	69.7
	MoHP, 2015 [42]	2015	10–14 years age group males in a national-based survey	57	57.5
	MoHP, 2015 [42]	2015	15–19 years age group males in a national-based survey	57	78.2
	MoHP, 2015 [42]	2015	20–24 years age group males in a national-based survey	35	68.4
	MoHP, 2015 [42]	2015	25–29 years age group males in a national-based survey	35	66.1
	MoHP, 2015 [42]	2015	30–34 years age group males in a national-based survey	19	72.8
	MoHP, 2015 [42]	2015	35–39 years age group males in a national-based survey	17	42.6
	MoHP, 2015 [42]	2015	40–44 years age group males in a national-based survey	16	78.0
	MoHP, 2015 [42]	2015	45–49 years age group males in a national-based survey	14	70.6
	MoHP, 2015 [42]	2015	50–54 years age group males in a national-based survey	14	67.1
	MoHP, 2015 [42]	2015	55–59 years age group males in a national-based survey	12	27.5
	Jhaveri, 2015 [59]	2012–14	Pregnant women/ANC attendees	98	55.0
	Kalil, 2010 [60]	2004–05	Children	121	72.0
	Kassem, 2000 [61]	1996	Pregnant women/ANC attendees	19	73.7
	Khamis, 2014 [62]	-	Pregnant women/ANC attendees	20	45.0
	Kumar, 1997 [63]	1994–96	Pregnant women/ANC attendees	65	31.0
	Nafeh, 2000 [64]	-	Participants in a population-based survey	514	63.0
	Strickland, 2002 [65]	-	Healthy individuals	99	74.7
	Tanaka,2004 [66]	1999	Blood donors	317	71.0
	Zuure,2013 [67]	2009–10	General population	11	90.9
**Iran**	Doosti, 2009 [68]	2003–04	Blood donors	76	62.0
	Farshadpour, 2010 [69]	2007–08	Blood donors	55	81.8
**Iraq**	Obied, 2014 [70]	2012–13	Blood donors	20	65.0
	Tawfeeq, 2013 [71]	2011–12	Blood donors	45	68.9
**Jordan**	Rashdan, 2008 [72]	2004–06	Blood donors	29	89.6
**Morocco**	Baha, 2013 [73]	2005–11	General population	195	70.9
	Benouda, 2009 [74]	2005–07	General population	158	39.2
**Pakistan**	Aziz, 2011 [75]	2005–09	Pregnant women/ANC attendees	640	79.7
	Donchuk, 2016 [76]	2015–16	Outpatient hospital attendees	1,107	89.0
	Idrees, 2008 [77]	1999–07	General population	857	49.2
	Idrees, 2008 [77]	1999–07	General population	141	50.4
	Karim, 2016 [78]	2015	Blood donors	60	93.0
	Khokhar, 2004 [79]	2001–02	Pregnant women/ANC attendees	18	72.0
	Rauf, 2011 [80]	2009	Refugees	18	44.4
	Rauf, 2011 [80]	2009	Refugees	34	50.0
**Palestine**	Shemer-Avni, 1998 [81]	-	Blood donors	34	71.0
	Shemer-Avni, 1998 [81]	-	Outpatient hospital attendees	11	64.0
**Tunisia**	Mejri, 2005 [82]	1996	General population	72	82.0
	Mejri, 2005 [82]	1996	General population	14	71.4
**Populations at intermediate risk (n = 20)**					
**Algeria**	Mouffok, 2013 [83]	2003–12	HIV infected patients	22	54.5
**Egypt**	Abdelwahab, 2012 [84]	2008–10	Diabetic patients	140	72.1
	Chehadeh, 2011 [85]	-	Diabetic patients	20	80.0
	Farghaly, 2014 [86]	-	Spouses of index patients	18	40.0
	El-Karaksy, 2010 [56]	2006–07	Health care workers	25	66.7
	Hassane, 1998 [87]	-	Household contacts of index patients	24	50.0
	Hassane, 1998[87]	-	Household contacts of index patients	21	59.1
	Hassane, 1998[87]	-	Household contacts of index patients	11	9.1
	Madwar, 1999 [88]	-	Prisoners	28	100
	Munier, 2013 [89]	2008–10	Diabetic patients	43	77.2
	Mohamed, 2013 [90]	-	Health care workers	79	51.2
	Shalaby, 2010 [91]	2007	Barbers and barbers’ clients	77	73.0
**Kuwait**	Chehadeh, 2011 [85]	-	Diabetic patients (Kuwaitis)	11	72.7
	Chehadeh, 2011 [85]	-	Diabetic patients (Egyptians)	20	80.0
**Lebanon**	Mahfoud, 2010 [92]	2007–08	Prisoners	12	50.0
**Libya**	Elzouki, 2014 [93]	2008–09	Health care workers	12	33.3
**Morocco**	Cacoub, 2000 [94]	1995–96	Inpatients and outpatients	60	75.0
	Rebbani, 2013 [95]	2006–10	HIV infected patients	27	77.8
**Pakistan**	Qureshi, 2007 [96]	-	Health care workers	21	76.0
	Zuberi, 2009 [97]	2004–08	Inpatients	10	90.0
**Populations at high risk of healthcare-related exposures (n = 51)**					
**Egypt**	Abdelwahab, 2012 [98]	-	Hemophilia patients	40	47.5
	Hussein, 2014 [99]	2007–08	Thalassemia patients	48	100
	Omar, 2011 [100]	-	Thalassemia patients	75	74.3
	Said, 2013 [101]	-	Thalassemia patients	47	100
	Salama, 2015 [102]	-	Thalassemia patients	40	55.0
**Iran**	Abdollahi, 2008 [103]	2003	Hemophilia patients	145	80.2
	Alvai, 2005 [104]	2002	Thalassemia patients	13	84.6
	Asguar, 2007 [105]	-	Hemophilia patients	21	80.9
	Assarehzadegan, 2012 [106]	2008–09	Hemophilia patients	47	89.4
	Azarkeivan, 2011 [107]	2008	Thalassemia patients	170	66.0
	Broumand, 2002 [108]	-	Hemodialysis patients	105	48.6
	Faranoush, 2006 [109]	-	Thalassemia patients	222	60.0
	Ghane, 2012 [110]	2010	Thalassemia patients	36	77.8
	Joukar, 2011 [111]	2009	Hemodialysis patients	61	50.8
	Kalantari, 2011 [112]	2009	Thalassemia patients	50	62.0
	Kalantari, 2011 [112]	2009	Hemophilia patients	495	70.1
	Makhlough, 2008 [113]	2006	Hemodialysis patients	39	53.8
	Mousavi, 2002 [114]	-	Thalassemia patients	22	77.3
	Samimi-Rad, 2007 [115]	2004	Bleeding disorder patients	34	68.0
	Samimi-Rad, 2008 [116]	2005	Hemodialysis patients	14	64.3
	Ziaee, 2005 [117]	2000	Thalassemia patients	44	56.8
**Iraq**	Al-Kubaisy, 2006 [118]	1998	Hemodialysis patients	50	76.0
	Al-Kubaisy, 2006 [118]	1998	Thalassemia patients	20	70.0
	Abdullah, 2012 [119]	2010	Hemophiliacs co-infected with HIV	92	26.1
	Khaled, 2014 [120]	2012	Thalassemia patients	50	88.0
	Shihab, 2014 [121]	2012–13	Hemodialysis patients	52	61.5
**Jordan**	Al-Sweedan, 2011 [122]	2008	Thalassemia patients	40	50.0
	Bdour, 2002 [123]	-	Hemodialysis patients	92	31.5
**Lebanon**	Abdelnour, 1997 [124]	-	Thalassemia patients	17	65.0
	Ramia, 2002 [125]	1999–00	Hemodialysis patients	55	34.5
**Libya**	Elzouki, 1995 [126]	-	Hemodialysis patients	32	72.0
**Morocco**	Benani, 1997 [127]	-	Hemodialysis patients	49	48.9
	Doblali, 2014 [128]	2010–12	Hemodialysis patients	26	65.4
	Foullous, 2015 [129]	-	Hemodialysis patients	194	54.1
	Lioussfi, 2014 [130]	2009	Hemodialysis patients	43	69.8
**Oman**	Al Naamani, 2015 [131]	1991–01	Thalassemia patients	65	51.0
**Palestine**	El-Ottol, 2010 [132]	2007	Hemodialysis patients	44	84.1
**Saudi Arabia**	Hussein, 1994 [133]	1993	Hemodialysis patients	27	70.4
**Syria**	Abdulkarim, 1998 [134]	-	Multi-transfused patients	56	87.5
	Yazaji, 2016 [135]	2012–13	Hemodialysis patients	18	22.2
**Tunisia**	Ayed, 2003 [136]	2001	Hemodialysis patients	310	75.5
	Ayed, 2003 [136]	2001	Hemodialysis patients	55	70.9
	Ayed, 2003 [136]	2001	Hemodialysis patients	44	90.9
	Ayed, 2003 [136]	2001	Hemodialysis patients	60	93.3
	Ayed, 2003 [136]	2001	Hemodialysis patients	243	60.5
	Ayed, 2003 [136]	2001	Hemodialysis patients	116	71.5
	Ben Othman, 2004 [137]	2000–02	Hemodialysis patients	42	76.2
	Ben Othman, 2004 [137]	2000–02	Hemodialysis patients	15	86.7
	Ben Othman, 2004 [137]	2000–02	Hemodialysis patients	33	78.8
	Hmaied, 2006 [138]	2001–03	Hemodialysis patients	79	73.0
	Sassi, 2000 [139]	-	Hemodialysis patients	27	51.8
**People who inject drugs (n = 5)**					
**Afghanistan**	Nasir, 2011 [140]	2006–08	People who inject drugs	165	58.2
	Nasir, 2011 [140]	2006–08	People who inject drugs	12	41.7
	Nasir, 2011 [140]	2006–08	People who inject drugs	44	59.1
**Iran**	Mansoori, 2003 [141]	1998–00	HIV patients with intravenous drug use as main mode of exposure	15	80.0
**Lebanon**	Mahfoud, 2010 [142]	2007–08	People who inject drugs	56	50.0
**Populations with liver-related conditions (n = 4)**					
**Algeria**	Bensalem, 2016 [143]	2012	Patients referred to a confirmatory laboratory test	3,204	66.2
**Algeria, Morocco, Tunisia**	Bahri, 2011 [144]	2002–05	Hepatocellular carcinoma patients	98	93.0
**Egypt**	Angelico, 1997 [145]	1993–95	Chronic liver disease patients	91	55.0
	Quinti, 1997 [146]	-	Acute viral hepatitis patients	23	87.0
	Strickland, 2002 [65]	-	Chronic liver disease patients	138	69.6
**Iraq**	Al-Kubaisy, 2014 [147]	2000–03	Hepatocellular carcinoma patients	17	70.8
**Morocco**	Tayeb, 2012 [148]	-	HCV Ab positive patients	46	43.5
**Pakistan**	Sundus, 2013 [149]	2009–10	Hepatitis patients	151	98.0
**Special clinical populations (n = 16)**					
**Egypt**	Cowgill, 2004 [52]	1999–03	Non-Hodgkin's lymphoma patients	106	89.0
	El Garf, 2012 [150]	2009	Rheumatoid arthritis patients	21	71.4
	Mahmoud, 2011 [151]	2009–10	Rheumatoid arthritis patients	22	63.6
	Mostafa, 2003 [152]	2000–01	Cancer patients on chemotherapy	13	38.5
	Mostafa, 2003 [152]	2000–01	Cancer patients on chemotherapy	44	47.7
	Sabry, 2005 [153]	-	Glomerulonephritis patients	90	55.6
	Sharaf-Eldeen, 2007 [154]	-	Lichen planus patients	43	76.8
	Youssef, 2009 [155]	-	Patients with liver complaints	156	57.7
**Pakistan**	Mahboob, 2003 [156]	1999–01	Lichen planus patients	16	62.5
**Saudi Arabia**	Halawani, 2012 [157]	2007–09	Urticarial patients	12	75.0
	Halawani, 2010 [158]	-	Lichen planus patients	24	62.5
**Tunisia**	Lakhoua Gorgi, 2010 [159]	1987–04	Renal transplant patients	24	91.7
**Mixed populations (n = 1)**					
**Libya**	Saleh, 1994 [40]	1992	Blood donors, health care workers, and outpatients	18	66.7

Abbreviations: Ab = Antibody, ANC = Antenatal care, HCV = Hepatitis C virus, MoHP = Ministry of Health and Population, RNA = Ribonucleic acid.

There were data on HCV viremic rate in 16 out of the 24 MENA countries (Table 1). Egypt contributed the largest number of data points (n = 89), and the majority of these were from studies in general populations.

### HCV viremic rate

HCV viremic rate varied across and within the risk populations with a broad range of 9–100% and a median of 68.8% (Table 2). The overall pooled mean HCV viremic rate (across all data points) was 67.6% (95% confidence interval (CI): 64.9–70.3%).

**Table 2:** Pooled mean estimate for hepatitis C virus (HCV) viremic rate by risk population in the Middle East and North Africa.

Population at risk	Studies	HCV Ab prevalence^a^	HCV RNA positivity among HCV Ab positive individuals					
			Sample	Prevalence	Prevalence	Heterogeneity measures		
	Total N	Mean (95% CI)	Total N	Range (%)	Mean (95% CI)	Q^b^ (p-value)	I^2^^c^ (95% CI)	Prediction interval^d^ (%)
General populations	81	10.4 (8.4–12.5)	10,448	26–100	66.9 (62.6–71.1)	1,510.9 (p < 0.0001)	94.7 (93.9–95.3)	29.1–95.3
Populations at intermediate risk	20	10.4 (6.8–14.6)	682	9–100	67.1 (58.6–75.2)	85.2 (p < 0.0001)	77.7 (66.0–85.4)	30.9–94.9
Populations at high risk healthcare-related exposures	51	31.3 (36.0–36.7)	3,814	22–100	68.5 (63.5–73.3)	478.0 (p < 0.0001)	89.5 (87.1–91.5)	33.7–94.8
People who inject drugs	5	42.2 (23.9–61.8)	292	50–80	57.4 (49.4–65.2)	5.6 (p = 0.23)	28.8 (0.0–72.3)	37.2–76.4
Populations with liver-related conditions	8	6.5 (43.8–83.0)	3,768	43–98	75.5 (61.0–87.6)	190.0 (p < 0.0001)	96.3 (94.5–97.5)	22.3–100
Special clinical populations	12	30.1 (19.6–41.8)	571	38–91	67.4 (56.7–77.3)	62.6 (p < 0.0001)	82.4 (70.6–89.5)	28.7–96.2
All studies^e^	**178**	**18.8 (16.7–21.1)**	**19,593**	**9–100**	**67.6 (64.9–70.3)**	**2,351.7 (p < 0.0001)**	**92.5 (91.6–93.2)**	**33.7–93.8**

^a^ This mean is the mean of HCV Ab prevalence in the study population from which the HCV viremic rate was extracted.

^b^ Q: The Cochran’s Q statistic is a measure assessing the existence of heterogeneity in effect size.

^c^ I^2^: A measure that assesses the magnitude of between-study variation that is due to differences in effect size across studies rather than chance.

^d^ Prediction interval: A measure that estimates the 95% interval in which the true effect size in a new study will lie.

^e^ A study including a mixed population group [40] was also considered in the meta-analysis.

Abbreviations: Ab = Antibody, CI = Confidence interval, HCV = Hepatitis C virus, RNA = Ribonucleic acid.

Across the risk populations (Table 2), the pooled mean HCV viremic rate was lowest at 57.4% (95% CI: 49.4–65.2%) in PWID, followed by 66.9% (95% CI: 62.6–71.1%) in the general populations, 67.1% (95% CI: 58.6–75.2%) in the populations at intermediate risk, 67.4% (95% CI: 56.7–77.3%) in the special clinical populations, 68.5% (95% CI: 63.5–73.3%) in the populations at high risk healthcare-related exposures, and 75.5% (95% CI: 61.0–87.6%) in populations with liver-related conditions.

Across countries or subregions (Table 3), the pooled mean HCV viremic rate was lowest at 62.1% (95% CI: 50.0–72.7%) in the Fertile Crescent, followed by 65.9% (95% CI: 55.3–75.9%) in the Gulf, 67.0% (95% CI: 63.1–70.8%) in Egypt, 68.6% (95% CI: 63.2–73.8%) in Iran, 70.4% (95% CI: 57.4–82.0%) in Afghanistan and Pakistan, and 70.4% (95% CI: 65.5–75.1%) in the Maghreb.

**Table 3:** Pooled mean estimate for hepatitis C virus (HCV) viremic rate by country or relevant subregion in the Middle East and North Africa.

Country or relevant subregion	Studies	HCV Ab prevalence^a^	HCV RNA positivity among HCV Ab positive individuals					
			Sample	Prevalence	Prevalence	Heterogeneity measures		
	Total N	Mean (95% CI)	Total N	Range (%)	Mean (95% CI)	Q ^b^ (p-value)	I^2^^c^ (95% CI)	Prediction interval^d^ (%)
Afghanistan/Pakistan	15	20 (13.1–27.9)	3,294	41–98	70.4 (57.4–82.0)	628.9 (p < 0.0001)	97.8 (97.2–98.3)	17.1–100
Egypt	89	16.9 (14.2–19.9)	8,348	9–100	67.0 (63.1–70.8)	1,108.0 (p < 0.0001)	92.1 (90.8–93.1)	31.5–94.4
Fertile Crescent	20	18.4 (13.2–24.2)	810	22–89	62.1 (50.0–72.7)	182.9 (p < 0.0001)	89.6 (85.4–92.6)	14.2–98.6
Gulf	6	20.5 (7.9–36.9)	159	51–80	65.9 (55.3–75.9)	7.9 (p = 0.15)	37.2 (0.0–75.0)	38.9–88.7
Iran	19	28.6 (16.1–43.0)	1,664	48–89	68.6 (63.2–73.8)	74.3 (p < 0.0001)	75.8 (62.3–84.4)	46.8–86.9
Maghreb	29	18.2 (13.9–22.9)	5,318	33–93	70.4 (65.5–75.1)	222.7 (p < 0.0001)	87.4 (83.1–90.7)	45.0–90.7
All countries	**178**	**18.8 (16.7–21.1)**	**19,593**	**9–100**	**67.6 (64.9–70.3)**	**2,351.7 (p < 0.0001)**	**92.5 (91.6–93.2)**	**33.7–93.8**

^a^ This mean is the mean of HCV Ab prevalence in the study population from which the HCV viremic rate was extracted.

^b^ Q: The Cochran’s Q statistic is a measure assessing the existence of heterogeneity in effect size.

^c^ I^2^: A measure that assesses the magnitude of between-study variation that is due to differences in effect size across studies rather than chance.

^d^ Prediction interval: A measure that estimates the 95% interval in which the true effect size in a new study will lie.

Abbreviations: Ab = Antibody, CI = Confidence interval, HCV = Hepatitis C virus, RNA = Ribonucleic acid.

Among the general populations (Table 4), the pooled mean HCV viremic rate was lowest among children (54.0%, 95% CI: 37.6–70.0%), and highest among blood donors (76.3%, 95% CI: 68.6–84.0%). Among populations at high risk of healthcare-related exposures, the pooled mean HCV viremic rate was lowest among hemodialysis patients (66.5%, 95% CI: 59.9–73.2%), and highest among hemophilia patients (73.6%, 95% CI: 63.9–82.3%)

**Table 4:** Pooled mean estimate for hepatitis C virus (HCV) viremic rate by risk subpopulation, sex, and sampling method in the Middle East and North Africa.

Variables	Studies	HCV Ab prevalence^a^	HCV RNA positivity among HCV Ab positive individuals					
			Sample	Prevalence	Effect size	Heterogeneity measures		
	Total N	Mean (95% CI)	Total N	Range (%)	Mean (95% CI)	Q^b^ (p-value)	I^2^^c^ (95% CI)	Prediction interval^d^ (%)
**Subpopulations among the general population**								
Blood donors	8	1.4 (0.5–2.7)	636	62–93	76.3 (68.6–84.0)	30.94 (p < 0.0001)	77.4 (55.2–88.6)	46.5–95.6
Children	7	3.7 (1.1–7.6)	225	27–75	54.0 (37.6–70.0)	26.1 (p < 0.0002)	77.0 (51.9–89.0)	7.6–96.4
Pregnant women/ANC attendees	9	7.7 (5.8–9.9)	1,084	26–79	58.1 (42.1–73.4)	149.3 (p < 0.0001)	94.6 (91.8–96.5)	7.4–99.2
Other general populations	57	13.9 (10.8–17.1)	8503	29–100	68.1 (62.9–73.1)	1,290.8 (p < 0.0001)	95.7 (94.9–96.3)	28.8–96.6
**Subpopulations among the populations at high risk healthcare-related exposures**								
Hemophilia patients	6	55.7 (35.6–74.9)	782	48–89	73.6 (63.9–82.3)	25.7 (p < 0.0001)	80.5 (58.0–91.0)	40.0–96.6
Hemodialysis patients	27	26.3 (22.0–30.1)	1,967	26–93	66.5 (59.5–73.2)	241.6 (p < 0.0001)	89.2 (85.6–92.0)	30.4–94.4
Thalassemia patients	16	29.6 (22.2–37.5)	1,027	34–100	72.1 (61.6–81.6)	177.5 (p < 0.0001)	91.5 (87.9–94.1)	26.3–99.9
**Sex**								
Females	33	11.0 (8.4–13.9)	2,712	26–100	65.4 (60.1–70.6)	225.5 (p < 0.0001)	85.8 (81.1–89.4)	36.3–89.6
Males	26	15.1 (10.4–20.5)	2,953	27–100	67.4 (58.1–76.0)	582.5 (p < 0.0001)	95.7 (94.6–96.6)	20.0–99.4
Mixed	119	22.5 (19.4–25.7)	13,928	9–100	68.2 (64.9–71.3)	1,542.1 (p < 0.0001)	92.3 (91.3–93.2)	34.9–93.9
**Sampling methods**								
Convenience sampling	126	22.4 (19.4–25.5)	13,713	9–100	68.2 (64.8–71.6)	1,843.8 (p < 0.0001)	93.2 (92.4–94.0)	31.5–95.6
National population-based and probability-based sampling	41	11.2 (7.9–14.9)	3,112	28–82	68.7 (66.6–70.8)	57.92 (p < 0.0003)	30.9 (0.0–53.1)	60.7–76.1
Other probability-based sampling	11	13.9 (5.9–24.5)	2,768	29–76	58.7 (49.4–67.6)	170.4 (p < 0.0001)	94.1 (91.3–96.0)	25.8–87.8

^a^ This mean is the mean of HCV Ab prevalence in the study population from which the HCV viremic rate was extracted.

^b^ Q: The Cochran’s Q statistic is a measure assessing the existence of heterogeneity in effect size.

^c^ I^2^: A measure that assesses the magnitude of between-study variation that is due to differences in effect size across studies rather than chance.

^d^ Prediction interval: A measure that estimates the 95% interval in which the true effect size in a new study will lie.

Abbreviations: Ab = Antibody, ANC = Antenatal care, CI = Confidence interval, HCV = Hepatitis C virus, RNA = Ribonucleic acid.

By sex (Table 4), the pooled mean HCV viremic rate was 65.4% (95% CI: 60.1–70.6%) among females, 67.4% (95% CI: 58.1–76.0%) among males, and 68.2% (95% CI: 64.9–71.3%) among the mixed-sex samples.

By sampling method (Table 4), the pooled mean HCV viremic rate was 58.7% (95% CI: 49.4–67.6%) for studies using probability-based sampling but not at the national level, 68.7% (95% CI: 66.6–70.8%) for studies using probability-based sampling at the national level, and 68.2% (95% CI: 64.8–71.6%) in studies using convenience sampling.

There was (overall) evidence for strong heterogeneity in HCV viremic rate in all the different meta-analyses with generally a p-value <0.0001 (Tables 2–4). The I^2^ for the pooled estimates indicated that the vast majority of the variation was due to true variation in HCV viremic rate across studies rather than chance (generally I^2^ >>50%). The prediction intervals were generally very broad confirming substantial variation in measured HCV viremic rate across studies. Forest plots for the meta-analyses by risk population can be found in S2 Fig.

### Predictors of HCV viremic rate and sources of heterogeneity

Table 5 displays the results of the univariable meta-regressions to identify the predictors of HCV viremic rate and sources of between-study heterogeneity. None of the hypothesized predictors were statistically significant (p-value >0.05), and none were eligible for inclusion in the final multivariable model (p-value >0.1). Therefore, no multivariable meta-regression was conducted.

**Table 5:** Univariable meta-regression models for hepatitis C virus (HCV) viremic rate in the Middle East and North Africa.

		Number of studies	Univariable analysis	
			OR (95% CI)	p-value
**Risk population**	General populations	81	1	-
	Populations at intermediate risk	20	0.9 (0.6–1.6)	0.887
	Populations of high risk healthcare-related exposures	51	1.1 (0.8–1.5)	0.483
	PWID	5	0.6 (0.3–1.7)	0.375
	Populations with liver-related conditions	8	1.7 (0.8–3.6)	0.147
	Special clinical populations	12	1.0 (0.5–1.8)	0.97
**Region**	Egypt	89	1	-
	Afghanistan/Pakistan	15	1.2 (0.7–2.1)	0.541
	Fertile Crescent^a^	20	0.8 (0.5–1.3)	0.309
	Gulf^b^	6	1.0 (0.4–2.3)	0.971
	Iran	19	1.1 (0.6–1.8)	0.793
	Maghreb^c^	29	1.1 (0.7–1.7)	0.566
**Sex**	Females	33	1	-
	Males	26	1.1 (0.7–1.9)	0.66
	Mixed	119	1.1 (0.7–1.6)	0.559
**Age**	Children	14	1	-
	Adults	164	1.5 (0.9–2.6)	0.139
**Prevalence of HCV Ab positive**	1–10%	56	1	-
	10–30%	69	1.2 (0.8–1.7)	0.331
	30–50%	32	1.1 (0.7–1.8)	0.637
	>50%	16	1.6 (0.9–2.8)	0.113
**Year of data collection**	Before 2000	42	1	-
	2000 and thereafter	136	1.2 (0.9–1.7)	0.217
**Sample size**	<50	89	1	-
	≥50	89	1.0 (0.7–1.4)	0.75
**Sampling methods**	Non-probability based sampling	126	1	-
	Probability based sampling	50	0.8 (0.6–1.2)	0.272

^a^ Fertile Crescent includes: Iraq, Jordan, Lebanon, Palestine, and Syria.

^b^ Gulf includes: Kuwait, Oman, and Saudi Arabia.

^c^ Maghreb includes: Algeria, Libya, Morocco, and Tunisia.

Abbreviations: Ab = Antibody, CI = Confidence interval, HCV = Hepatitis C virus, PWID = People who inject drugs, OR = Odds ratio.

Though no variables were significantly predictive of HCV viremic rate, there were notably trends of lower viremic rate for females and children (Table 5), and trends of higher viremic rate for populations with liver-related conditions and for populations with high (>50%) HCV Ab prevalence.

## Discussion

Through a comprehensive analysis of systematically extracted data, we investigated HCV viremic rate in the MENA region. We found that about two-thirds of HCV Ab positive individuals are chronically infected with HCV infection. Though the viremic rate varied widely across studies, the pooled mean HCV viremic rate was similar regardless of risk population or subpopulation, country or subregion, HCV Ab prevalence, sex, or study sampling method. The overall pooled mean viremic rate of 67.6% (95% CI: 64.9–70.3%) was also similar to that found in large population-based and nationally-representative surveys such as those of the Demographic and Health Surveys in Egypt that reported a viremic rate of 66.6% (in 2008) [41] and 70.2% (in 2015) [42].

HCV viremic rate is defined as the proportion of *chronically infected* individuals (HCV Ab positive and RNA positive), out of all *ever infected* individuals (HCV Ab positive regardless of RNA status). Accordingly, it is closely linked to HCV clearance rate, defined as the proportion of people who spontaneously clear their infection—that is the proportion of people who clear their *acute infection* and do not become *chronically infected* [11]. Our results then imply that over 30% of infected individuals spontaneously clear their infection, a higher proportion than that estimated in prospective cohort studies of about 25% [7, 23]. While prospective studies provide a direct approach to estimating the clearance rate, there are known methodological limitations and potential biases that may lead to underestimation of clearance rate [7, 11, 22]. Our results, using an independent methodology from that of prospective studies, suggest that one-third of infected individuals spontaneously clear their infection.

In planning for this study, our implied hypothesis was that we will identify several predictors of higher HCV viremic rate. PWID and populations at high risk of healthcare-related exposures may have a weaker immune system and are at a higher risk of HCV reinfection, therefore should have a higher viremic rate. Female sex is associated with higher spontaneous clearance rate [7, 23, 43], and therefore we expected the viremic rate among men to be larger than that among women. We further expected a higher viremic rate in populations with higher HCV Ab prevalence, as HCV Ab prevalence can be seen as a proxy for the risk of repeated HCV exposures. Lastly, we expected a higher viremic rate in populations with liver-related conditions, since the presence of these conditions could be indicative of chronic HCV infection.

Nevertheless, none of these hypothesized effects were identified as statistically significant in our meta-regression analyses. Though there was a trend of lower viremic rate in women-only studies, and trends of higher viremic rate in populations with liver-related conditions and populations with higher (>50%) HCV Ab prevalence, none of these trends reached statistical significance. These results suggest that either some of these effects may not be present as originally hypothesized, or that the effect size of these effects was not large enough to be detected in our sample of 178 viremic rate measures, or that the heterogeneity in the effect size lowered the power of the analysis to detect these differences.

Though we could not identify any significant predictor of HCV viremic rate, the viremic rate varied widely across studies. This may suggest that much of this variation could be due to random effects, such as those related to the complex laboratory methods used in assessing the viremic rate. Assessment of HCV viremic rate requires a two-test algorithm, for HCV Ab and HCV RNA, and the diagnostic assays and protocols can vary from one study to another. Different assays, whether for HCV Ab or for HCV RNA, may also have different sensitivities and specificities, which can impact the estimated HCV viremic rate [44, 45]. Even small random errors in assessing the denominator (HCV Ab positive cases), or the numerator (HCV RNA positive cases), can lead to large variation in calculated viremic rate.

Another source of random errors in calculated HCV viremic rate could be sampling variation as the “effective” sample size in viremic rate studies (the number of HCV Ab positive cases), tend to be small (Table 1). The number of HCV Ab positive cases is most often a *subsample* of the original study sample size—the original study sample size is the number of individuals recruited in the original study whose serostatus could be HCV Ab negative or HCV Ab positive. The median size of the (sub) sample of HCV Ab positive cases in included studies was only 50. With a viremic rate of 67.6% (as was the pooled estimate), this small median sample size leads to a wide confidence interval (95% CI: 53.3%-80.5%). This highlights how (sub) sample size could be a major cause of the observed variation.

The large variations in HCV viremic rate across studies (Table 1), but the small variations in the pooled mean HCV viremic rates (Tables 2–4), suggest caution against using the highly variable and possibly error-prone study-specific viremic rates in estimations of the number of HCV chronic carriers in different populations and countries, instead of the stable pooled means. We advocate here for the use of one standardized HCV viremic rate, say the overall pooled mean estimated in this study (Table 2), in ongoing chronic HCV infection estimations—such as the global estimations being conducted for the World Health Organization [3]. We further advocate for the use of pooled means, rather than study-specific estimates, for assessing and monitoring the progress in (and coverage of) HCV treatment programs, as we forge ahead towards HCV elimination by 2030. Of notice here that this progress monitoring will require repeated population-based measures of HCV antibody positivity and HCV RNA positivity in the same population, with sufficiently large sample sizes to assess statistically the trends in HCV viremic rate.

Our study has several limitations. The availability of data varied by risk population and country, and we did not identify any HCV viremic rate data for eight MENA countries. The number of studies was limited for some risk populations—only five studies were identified for PWID, and these were mostly conducted among PWID with access to prevention programs. Sample size varied across studies, and the sampled risk population may not have been representative of the wider risk population in the country. Despite these limitations, we identified a substantial volume of viremic rate data in MENA that facilitated the conduct of different types of analyses, thereby generating informative inferences.

## Conclusions

Two-thirds of HCV Ab positive individuals in MENA are chronically infected with HCV infection, implying that over 30% of infected individuals spontaneously clear their infection. Though there was extensive variation in the study-specific HCV viremic rates, the pooled mean viremic rates were similar regardless of risk population or subpopulation, country or subregion, HCV Ab prevalence in the background population, or sex. These findings argue for the use of one standardized HCV viremic rate, such as the overall pooled mean viremic rate provided in this study, in estimations of the number of HCV chronic carriers in different populations and countries. These findings also highlight the utility of using the pooled mean viremic rate as an indicator to track the progress in (and coverage of) HCV treatment programs in different risk populations and countries, as viral hepatitis treatment programs are established and/or expanded with the ultimate target of HCV elimination by 2030.

## Supporting information

S1 Fig Preferred Reporting Items for Systematic Reviews and Meta-analyses (PRISMA) checklist. (DOCX)

S2 Fig Forest plots presenting the outcomes for the pooled mean hepatitis C virus (HCV) viremic rate by risk population in the Middle East and North Africa. (DOCX)

S1 Box PubMed search strategies for systematically reviewing hepatitis C virus (HCV) in the Middle East and North Africa. (DOCX)

S2 Box Embase search strategies for systematically reviewing hepatitis C virus (HCV) in the Middle East and North Africa. (DOCX)
