# Supplementary material for: Hepatitis C virus viremic rate in the Middle East and North Africa: Systematic synthesis, meta-analyses, and meta-regressions
Source: PLoS One. 2017 Oct 31;12(10):e0187177. doi: 10.1371/journal.pone.0187177 (PMC5663443; doi:10.1371/journal.pone.0187177)
Supplement: S1 Fig — (DOCX) [file pone.0187177.s001.docx]

**S1 Fig**. Preferred Reporting Items for Systematic Reviews and Meta-analyses (PRISMA) checklist [1].

| **Section/topic** | **#** | **Checklist item** | **Reported on page #** |
| --- | --- | --- | --- |
| **TITLE** | | |  |
| Title | 1 | Identify the report as a systematic review, meta-analysis, or both. | 1 |
| **ABSTRACT** | | |  |
| Structured summary | 2 | Provide a structured summary including, as applicable: background; objectives; data sources; study eligibility criteria, participants, and interventions; study appraisal and synthesis methods; results; limitations; conclusions and implications of key findings; systematic review registration number. | 2 |
| **INTRODUCTION** | | |  |
| Rationale | 3 | Describe the rationale for the review in the context of what is already known. | 4-5 |
| Objectives | 4 | Provide an explicit statement of questions being addressed with reference to participants, interventions, comparisons, outcomes, and study design (PICOS). | 5 |
| **METHODS** | | |  |
| Protocol and registration | 5 | Indicate if a review protocol exists, if and where it can be accessed (e.g., Web address), and, if available, provide registration information including registration number. | 6, [2-10] |
| Eligibility criteria | 6 | Specify study characteristics (e.g., PICOS, length of follow-up) and report characteristics (e.g., years considered, language, publication status) used as criteria for eligibility, giving rationale. | 7, [2-10] |
| Information sources | 7 | Describe all information sources (e.g., databases with dates of coverage, contact with study authors to identify additional studies) in the search and date last searched. | 6-7, [2-10] |
| Search | 8 | Present full electronic search strategy for at least one database, including any limits used, such that it could be repeated. | 6, S1 Box, S2 Box, [2-10] |
| Study selection | 9 | State the process for selecting studies (i.e., screening, eligibility, included in systematic review, and, if applicable, included in the meta-analysis). | 7, [2-10] |
| Data collection process | 10 | Describe method of data extraction from reports (e.g., piloted forms, independently, in duplicate) and any processes for obtaining and confirming data from investigators. | [2-10] |
| Data items | 11 | List and define all variables for which data were sought (e.g., PICOS, funding sources) and any assumptions and simplifications made. | [2-10] |
| Risk of bias in individual studies | 12 | Describe methods used for assessing risk of bias of individual studies (including specification of whether this was done at the study or outcome level), and how this information is to be used in any data synthesis. | [2-10] |
| Summary measures | 13 | State the principal summary measures (e.g., risk ratio, difference in means). | 8 |
| Synthesis of results | 14 | Describe the methods of handling data and combining results of studies, if done, including measures of consistency (e.g., I^2^) for each meta-analysis. | 8, [2-10] |

**References:**

1. Moher D, Liberati A, Tetzlaff J, Altman DG, Group P. Preferred reporting items for systematic reviews and meta-analyses: the PRISMA statement. PLoS med. 2009;6(7):e1000097.

2. Al-Kanaani Z KS, Abu-Raddad LJ. . The epidemiology of hepatitis C virus in Pakistan: systematic review and meta-analyses (under preparation). 2017.

3. Chaabna K, Kouyoumjian SP, Abu-Raddad LJ. Hepatitis C virus epidemiology in Djibouti, Somalia, Sudan, and Yemen: systematic review and meta-analysis. PloS one. 2016;11(2):e0149966.

4. Chaabna K, Mohamoud YA, Chemaitelly H, Mumtaz GR, Abu-Raddad LJ. Protocol for a systematic review and meta-analysis of hepatitis C virus (HCV) prevalence and incidence in the Horn of Africa sub-region of the Middle East and North Africa. Systematic reviews. 2014;3(1):146.

5. Chemaitelly H, Chaabna K, Abu-Raddad LJ. The epidemiology of hepatitis C virus in the Fertile Crescent: systematic review and meta-analysis. PloS one. 2015;10(8):e0135281.

6. Chemaitelly H, Mahmud S, Rahmani AM, Abu-Raddad LJ. The epidemiology of hepatitis C virus in Afghanistan: Systematic review and meta-analysis. International Journal of Infectious Diseases. 2015;40:54-63.

7. Fadlalla FA, Mohamoud YA, Mumtaz GR, Abu-Raddad LJ. The epidemiology of hepatitis C virus in the Maghreb region: systematic review and meta-analyses. PloS one. 2015;10(3):e0121873.

8. Mohamoud YA, Riome S, Abu-Raddad LJ. Epidemiology of hepatitis C virus in the Arabian Gulf countries: Systematic review and meta-analysis of prevalence. International Journal of Infectious Diseases. 2016;46:116-25.

9. Mahmud S, Akbarzadeh V, Abu-Raddad L. The epidemiology of hepatitis C virus in Iran: Systematic review and meta-analyses (under review). 2017.

10. Kouyoumjian SP, Chemaitelly H, Abu-Raddad LJ. Characterizing hepatitis C virus epidemiology in Egypt: systematic reviews, meta-analyses, and meta-regressions (under review). 2017.
