# Supplementary material for: Hepatitis C virus viremic rate in the Middle East and North Africa: Systematic synthesis, meta-analyses, and meta-regressions
Source: PLoS One. 2017 Oct 31;12(10):e0187177. doi: 10.1371/journal.pone.0187177 (PMC5663443; doi:10.1371/journal.pone.0187177)

**S2 Fig.** Forest plots presenting the outcomes for the pooled mean hepatitis C virus (HCV) viremic rate by risk population in the Middle East and North Africa.

1. General populations


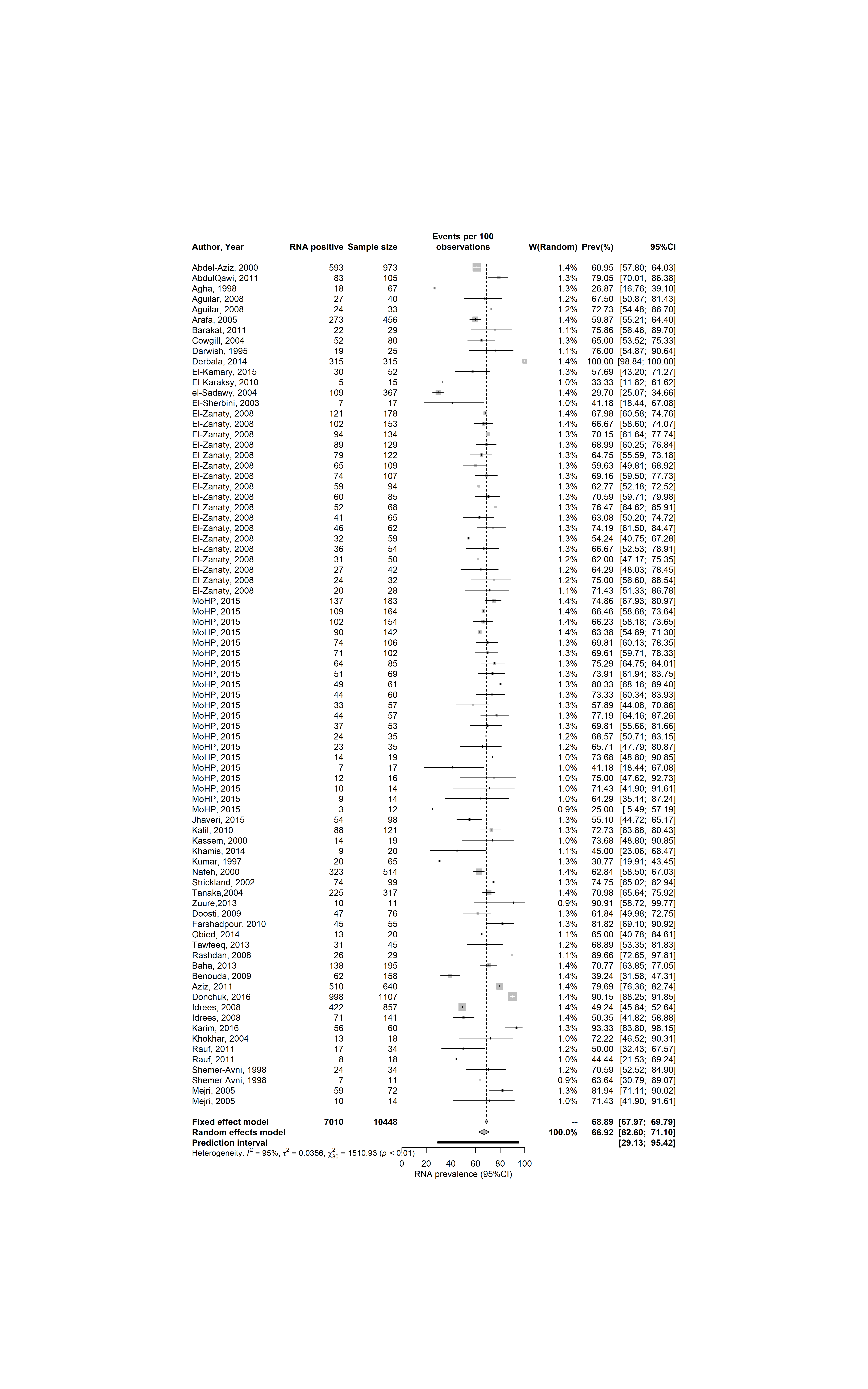


1. Populations at intermediate risk
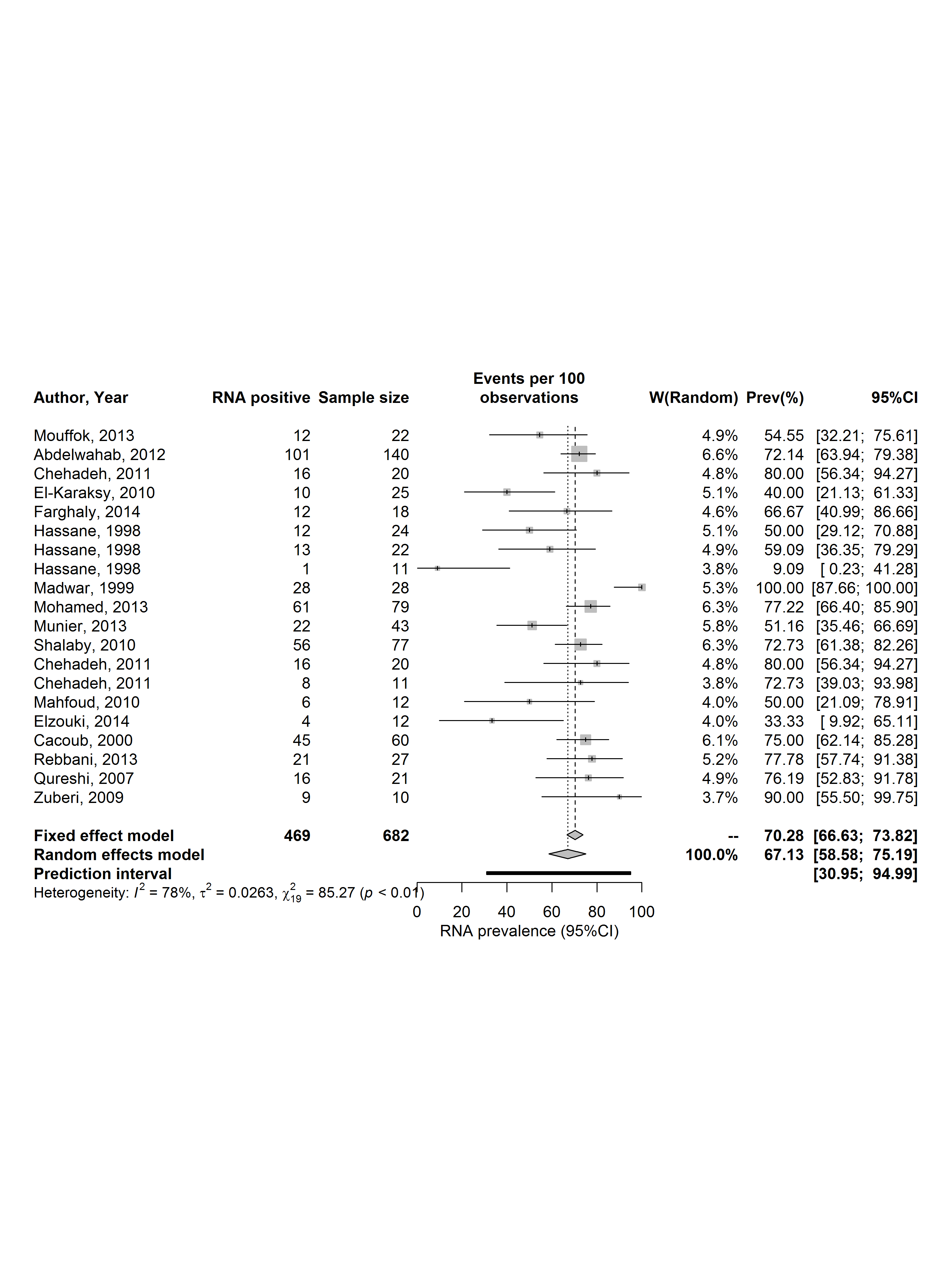

2. Populations at high risk of healthcare-related exposures


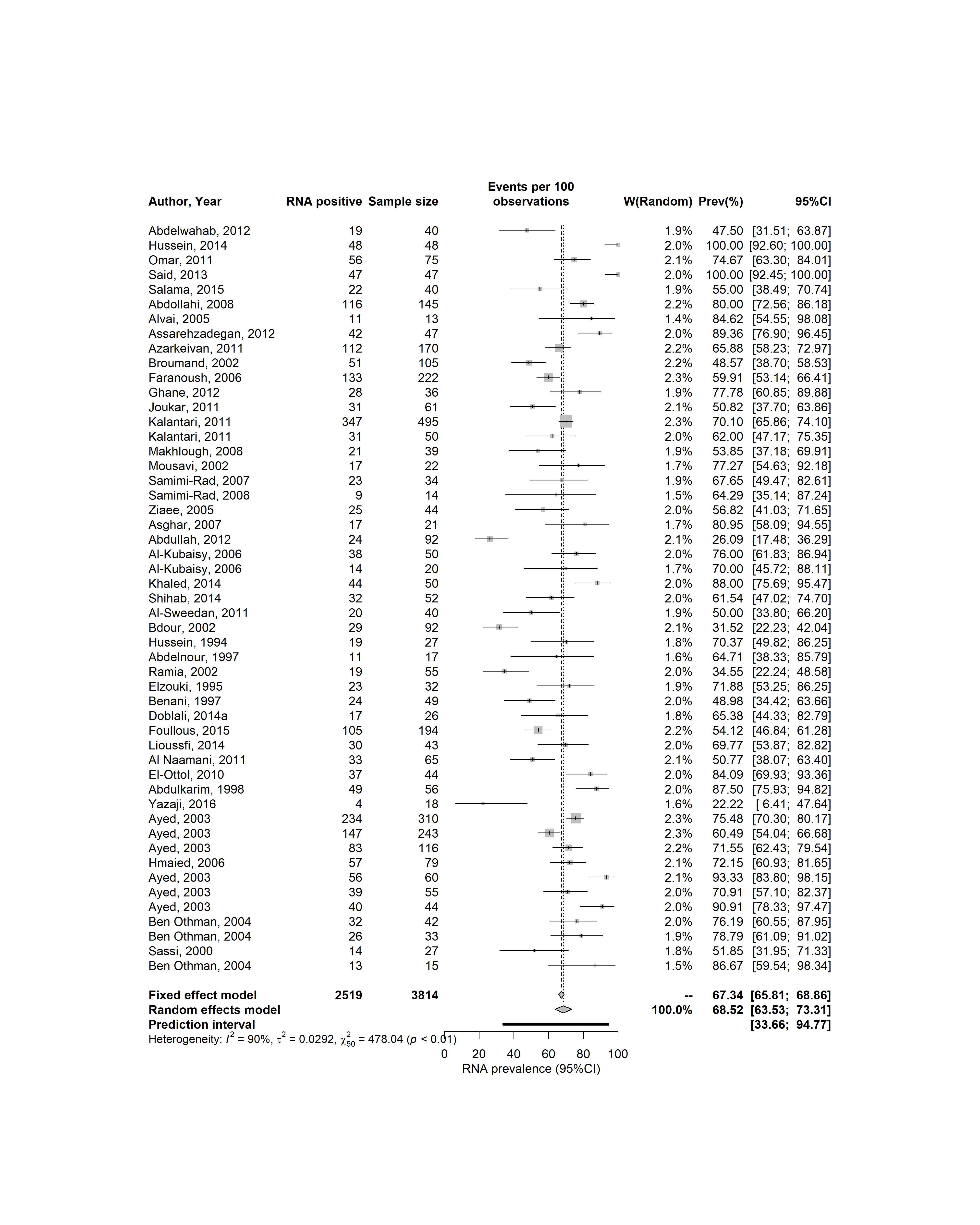


1. People who inject drugs


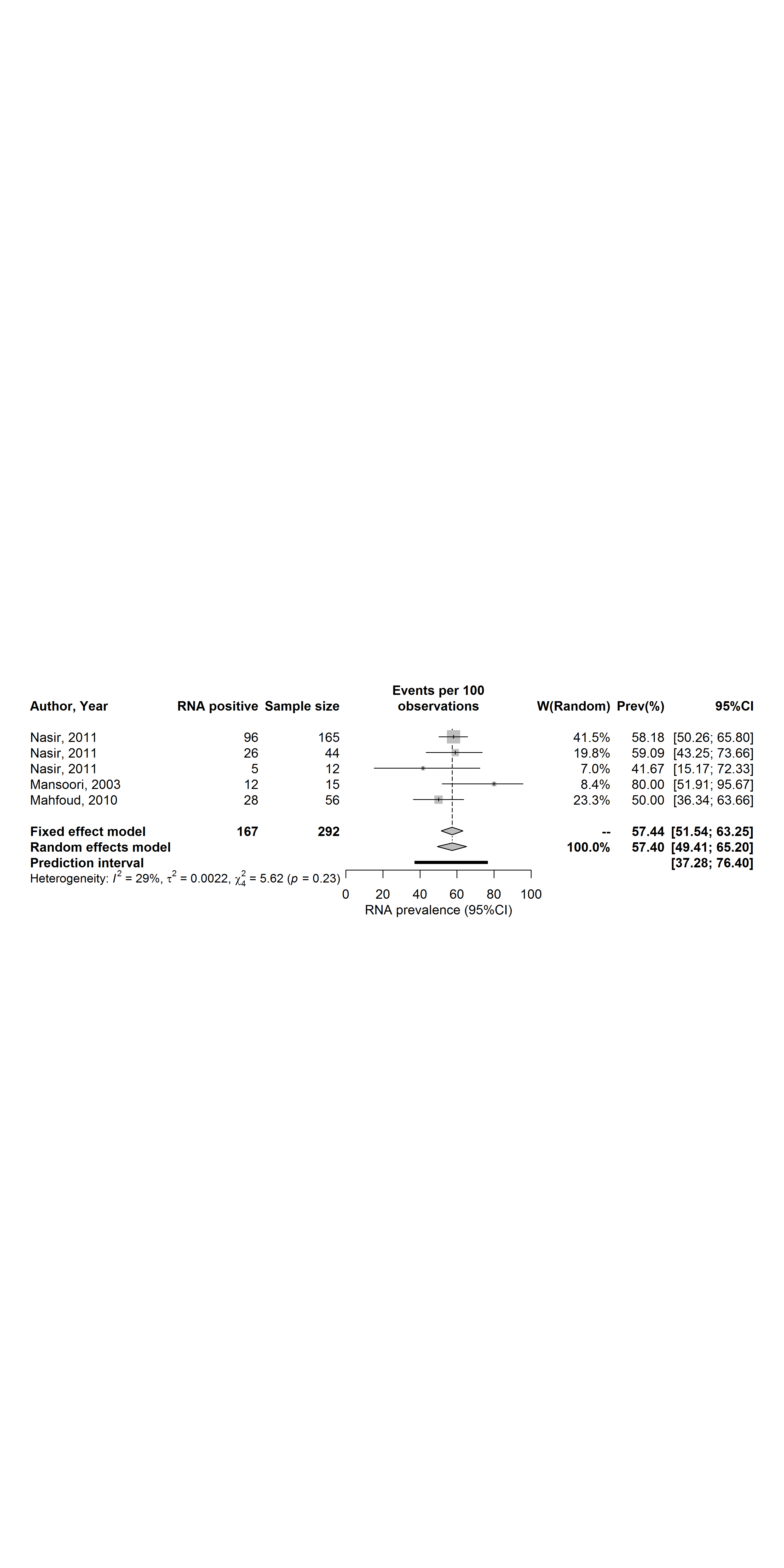


1. Populations with liver-related conditions


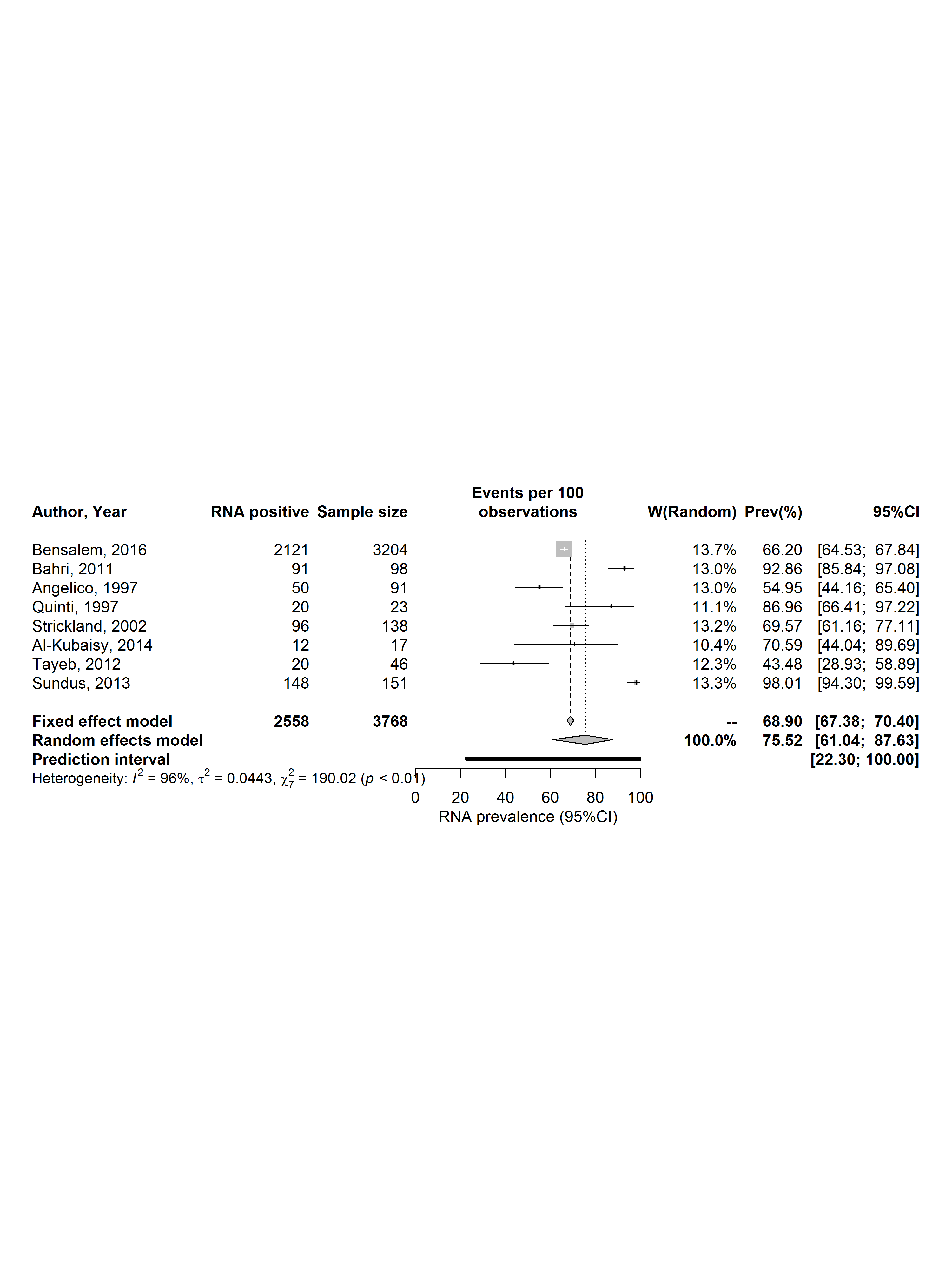


1. Special clinical populations


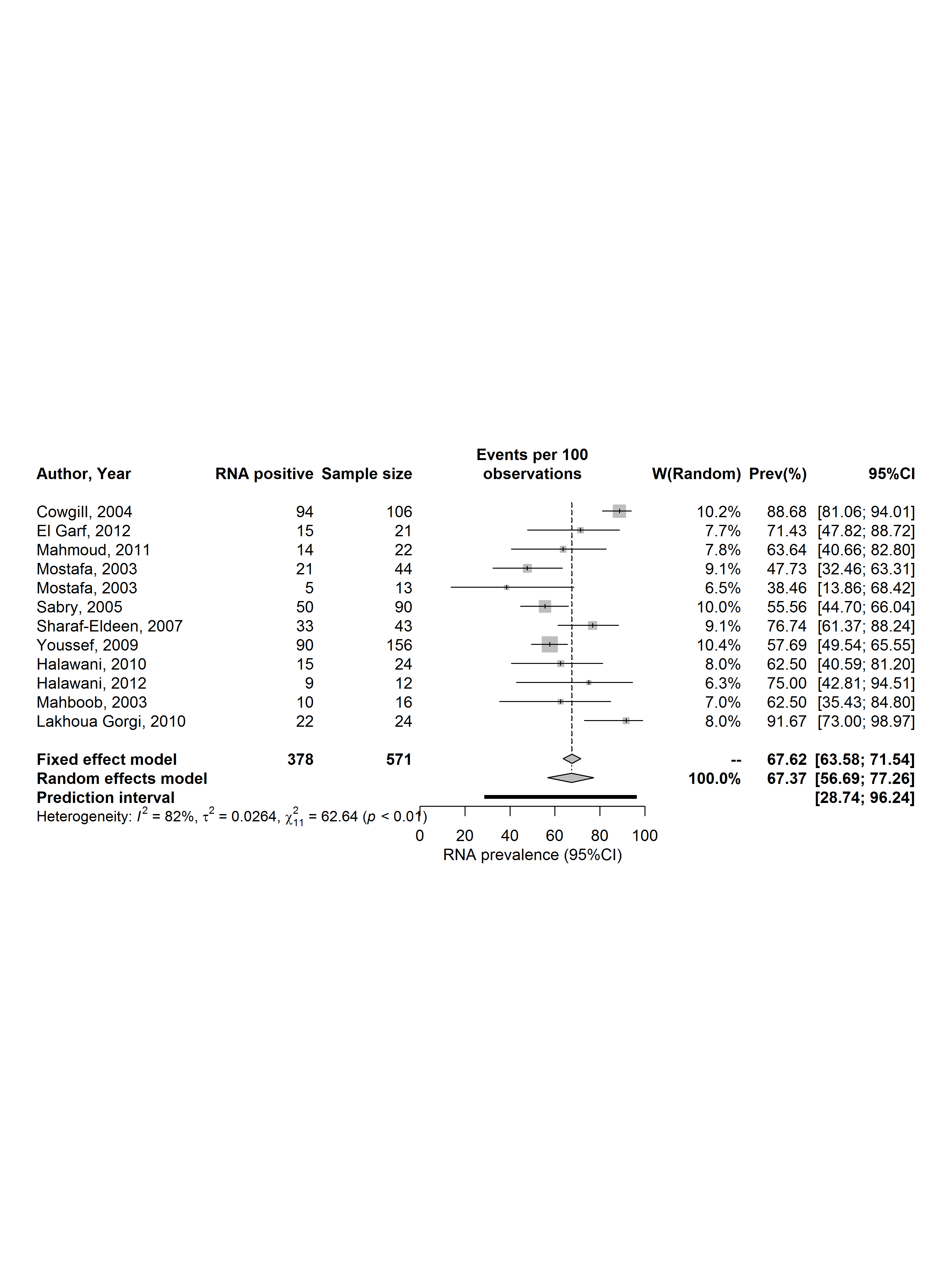

Supplement: S2 Fig — (DOCX) [file pone.0187177.s002.docx]
