# Supplementary material for: Hepatitis C virus viremic rate in the Middle East and North Africa: Systematic synthesis, meta-analyses, and meta-regressions
Source: PLoS One. 2017 Oct 31;12(10):e0187177. doi: 10.1371/journal.pone.0187177 (PMC5663443; doi:10.1371/journal.pone.0187177)
Supplement: S1 Box — (DOCX) [file pone.0187177.s003.docx]

**S1 Box.** PubMed search strategies for systematically reviewing hepatitis C virus (HCV) epidemiology in the Middle East and North Africa.

| **Systematic review of HCV in Egypt [1, 2]**  ("Hepatitis C"[Mesh] OR "Hepacivirus"[Mesh] OR "Hepatitis C, Chronic"[Mesh] OR "Hepatitis C Antibodies"[Mesh] OR "Hepatitis C Antigens"[Mesh] OR "HCV"[Text] OR “Hepatitis C”[Text]) AND (“Egypt” [Text] OR “Egyptian” [Text] OR “Egyptians” [Text]).  **Systematic review of HCV in the Fertile Crescent region (Iraq, Jordan, Lebanon, Palestine, and Syria) [3]**  ("Hepatitis C"[Mesh] OR "Hepacivirus"[Mesh] OR "Hepatitis C, Chronic"[Mesh] OR "Hepatitis C Antibodies"[Mesh] OR "Hepatitis C Antigens"[Mesh] OR "HCV"[Text] OR “Hepatitis C”[Text]) AND ( “Iraq” [Text] OR “Iraqi”[Text] OR “Jordan” [Text] OR “Jordanian”[Text] OR “Lebanon”[Text] OR “Lebanese”[Text] OR “Syria”[Text] OR “Syrian”[Text] OR “Palestine”[Text] OR “West Bank”[Text] OR “Gaza”[Text] OR “Palestinian”[Text]).  **Systematic review of HCV in Afghanistan [3]**  ("Hepatitis C"[Mesh] OR "Hepacivirus"[Mesh] OR "Hepatitis C, Chronic"[Mesh] OR "Hepatitis C Antibodies"[Mesh] OR "Hepatitis C Antigens"[Mesh] OR "HCV"[Text] OR “Hepatitis C”[Text] OR “Hepacivirus”[Text]) AND (“Afghanistan”[Mesh] OR “Afghanistan” [Text] OR “Afghan” [Text])  **Systematic review of HCV in the Maghreb region (Algeria, Libya, Mauritania, Morocco, and Tunisia) [4]**  ("Hepatitis C"[Mesh] OR "Hepatitis C Antibodies"[Mesh] OR "Hepatitis C Antigens"[Mesh] OR “Hepacivirus”[Mesh] OR “Hepatitis C, chronic/epidemiology”[Mesh] OR “Hepatitis C, chronic/etiology”[Mesh] OR “Hepatitis C, chronic/transmission”[Mesh] OR “Hepatitis C, chronic/virology”[Mesh] OR “Hepatitis C”[Text] OR “HCV”[Text]) AND ((“Africa,Northern” [Mesh] or “Algeria” [Mesh] or “Libya” [Mesh] or “Morocco” [Mesh] or “Tunisia” [Mesh] or “Mauritania” [Mesh] or “Algeria” [Text] or “Libya” [Text] or “Morocco” [Text] or “Tunisia” [Text] or “Mauritania” [Text] or “Algeria*” [Text] or “Libya*” [Text] or “Moroccan*” [Text] or “Tunis*” [Text] or “Mauritania*” [Text] or “North Africa” [Text] or “North-Africa” [Text] or (“Africa” [Text] AND “Northern” [Text]) or “Northern Africa” [Text] or “Maghreb” [Text] or “Maghrib”[Text])  **Systematic review of HCV in the Arabian Gulf region (Bahrain, Kuwait, Oman, Qatar, Saudi Arabia, and United Arab Emirates) [5]**  ("Hepatitis C"[Mesh] OR "Hepatitis C Antibodies"[Mesh] OR "Hepatitis C Antigens"[Mesh] OR “Hepacivirus”[Mesh] OR “Hepatitis C, chronic/epidemiology”[Mesh] OR “Hepatitis C, chronic/etiology”[Mesh] OR “Hepatitis C, chronic/transmission”[Mesh] OR “Hepatitis C, chronic/virology”[Mesh] OR “Hepatitis C”[Text] OR “HCV”[Text]) AND (("Saudi Arabia"[Mesh] or "Kuwait"[Mesh] or "Bahrain"[Mesh] or "Oman"[Mesh] or "United Arab Emirates"[Mesh] or "Qatar"[Mesh] or “Gulf”[Text] or “Saudi*”[Text] or “United Arab Emirates”[Text] or “U.A.E” [Text] or “UAE” [Text] or “Emirat*” [Text] or “Oman*” [Text] or “Kuwait*”[Text] or “Bahrain*”[Text] or “Qatar*”[Text]))  **Systematic review of HCV in Djibouti, Somalia, Sudan, and Yemen [6]**  ("Hepatitis C"[Mesh] OR "Hepatitis C Antibodies"[Mesh] OR "Hepatitis C Antigens"[Mesh] OR "Hepacivirus"[Mesh] OR "Hepatitis C, chronic/epidemiology"[Mesh] OR "Hepatitis C, chronic/etiology"[Mesh] OR "Hepatitis C, chronic/transmission"[Mesh] OR "Hepatitis C, chronic/virology"[Mesh] OR "Hepatitis C"[Text] OR "HCV"[Text] OR "Hepatite"[Text] OR "VHC"[Text] OR "HVC"[Text]) AND ("Yemen"[Mesh] OR "Djibouti"[Mesh] OR "Somalia"[Mesh] OR "Sudan"[Mesh] OR "Africa"[Mesh] OR "Yemen"[Text] OR “Yemeni” [Text] OR "Djibouti"[Text] OR "Somalia"[Text] OR “Somali” [Text] OR "Sudan"[Text] OR “Sudanese” [Text] OR " Africa"[Text] OR “African” [Text])  **Systematic review of HCV in Iran [7]**  ("Hepatitis C"[Mesh] OR "Hepatitis C Antibodies"[Mesh] OR "Hepatitis C Antigens"[Mesh] OR "Hepacivirus"[Mesh] OR "Hepatitis C"[Text] OR "Hepacivirus"[Text] OR "HCV"[Text]) AND ("Iran"[Mesh] or Iran*[text] or "Persia"[Mesh] or Persia*[text])  **Systematic review of HCV in Pakistan [8]**  ("Hepatitis C"[Mesh] OR "Hepatitis C Antibodies"[Mesh] OR "Hepatitis C Antigens"[Mesh] OR "Hepacivirus"[Mesh] OR "Hepatitis C"[Text] OR "Hepacivirus"[Text] OR "HCV"[Text]) AND ("Pakistan"[Mesh] or Pakistan*[text]) |
| --- |

**References:**

1. Mohamoud YA, Mumtaz GR, Riome S, Miller D, Abu-Raddad LJ. The epidemiology of hepatitis C virus in Egypt: a systematic review and data synthesis. BMC infectious diseases. 2013;13(1):288.

2. Kouyoumjian SP, Chemaitelly H, Abu-Raddad LJ. Characterizing hepatitis C virus epidemiology in Egypt: systematic reviews, meta-analyses, and meta-regressions (under review). 2017.

3. Chemaitelly H, Chaabna K, Abu-Raddad LJ. The epidemiology of hepatitis C virus in the Fertile Crescent: systematic review and meta-analysis. PloS one. 2015;10(8):e0135281.

4. Fadlalla FA, Mohamoud YA, Mumtaz GR, Abu-Raddad LJ. The epidemiology of hepatitis C virus in the Maghreb region: systematic review and meta-analyses. PloS one. 2015;10(3):e0121873.

5. Mohamoud YA, Riome S, Abu-Raddad LJ. Epidemiology of hepatitis C virus in the Arabian Gulf countries: Systematic review and meta-analysis of prevalence. International Journal of Infectious Diseases. 2016;46:116-25.

6. Chaabna K, Kouyoumjian SP, Abu-Raddad LJ. Hepatitis C virus epidemiology in Djibouti, Somalia, Sudan, and Yemen: systematic review and meta-analysis. PloS one. 2016;11(2):e0149966.

7. Mahmud S, Akbarzadeh V, Abu-Raddad L. The epidemiology of hepatitis C virus in Iran: Systematic review and meta-analyses (under review). 2017.

8. Al-Kanaani Z MS, Abu-Raddad L. The epidemiology of hepatitis C virus in Pakistan: systematic review and meta-analyses (under preparation). 2017.
