# Supplementary material for: Hepatitis C virus viremic rate in the Middle East and North Africa: Systematic synthesis, meta-analyses, and meta-regressions
Source: PLoS One. 2017 Oct 31;12(10):e0187177. doi: 10.1371/journal.pone.0187177 (PMC5663443; doi:10.1371/journal.pone.0187177)
Supplement: S2 Box — (DOCX) [file pone.0187177.s004.docx]

**S2 Box.** Embase search strategies for systematically reviewing hepatitis C virus (HCV) epidemiology in the Middle East and North Africa.

| **Systematic review of HCV in Egypt [1, 2]**  (exp hepatitis C/ or exp Hepatitis C virus/ or hepatitis C.mp. or HCV.mp. or hepacivirus.mp.) And (Egypt.mp. or Egyptian.mp. or Egyptians.mp.)  **Systematic review of HCV in the Fertile Crescent region (Iraq, Jordan, Lebanon, Palestine, and Syria) [3]**  (exp hepatitis C/ or exp Hepatitis C virus/ or hepatitis C.mp. or HCV.mp. or hepacivirus.mp.) And (Iraq.mp. or exp Iraq/ or Iraqi.mp. or Syria.mp. or exp Syrian Arab Republic/ or Syrian.mp. or Jordan.mp. or exp Jordan/ or Jordanian.mp. or Lebanon.mp. or exp Lebanon/ or Lebanese.mp. or Palestine.mp. or exp Palestine/ or Palestinian.mp. or West Bank.mp. or Gaza.mp.)  **Systematic review of HCV in Afghanistan [4]**  (exp hepatitis C/ or exp Hepatitis C virus/ or exp hepatitis C antibody/ or exp hepatitis C antigen/ or hepatitis C.mp. or HCV.mp.) And (Afghanistan.mp. or exp Afghanistan/ or Afghan.mp. or exp Afghan/)  **Systematic review of HCV in the Maghreb region (Algeria, Libya, Mauritania, Morocco, and Tunisia) [5]**  (exp North Africa/ or North Africa.mp. or Maghreb.mp. or Algeria*.mp. or exp Algeria/ or Libya*.mp. or exp Libya/ or Morocc*.mp. or Morocco*.mp. or Moroccan*.mp. or exp Morocco/ or Tunisia*.mp. or exp Tunisia/ or Mauritania*.mp. or exp Mauritania/) AND (exp hepatitis C/ or exp Hepatitis C virus/ or hepatitis C.mp. or HCV.mp. or hepacivirus.mp.)  **Systematic review of HCV in the Arabian Gulf region (Bahrain, Kuwait, Oman, Qatar, Saudi Arabia, and United Arab Emirates) [6]**  (Qatar*.mp. or exp Qatar/ or Kuwait*.mp. or exp Kuwait/ or Saudi*.mp. or exp Saudi Arabia/ or Bahrain*.mp. or exp Bahrain/ or Oman*.mp. or exp Oman/ or United Arab Emirates.mp. or exp United Arab Emirates/ or Emirat*.mp. or UAE.mp. or exp dubai/ or dubai.mp.) AND (exp hepatitis C/ or exp Hepatitis C virus/ or hepatitis C.mp. or HCV.mp. or hepacivirus.mp.)  **Systematic review of HCV in Djibouti, Somalia, Sudan, and Yemen [7]**  (Yemen*.mp. or exp Yemen/ or Djibouti.mp. or exp Djibouti/ or Somali*.mp. or exp Somalia/ or Sudan*.mp. or exp Sudan/ or Africa*.mp. or exp Africa/) AND (exp hepatitis C/ or exp Hepatitis C virus/ or hepatitis C.mp. or HCV.mp. or hepacivirus.mp. or Hepatite.mp. or VHC.mp. or HVC.mp.)  **Systematic review of HCV in Iran [8]**  (exp Iran/ OR Iran*.mp. OR Persia*.mp.) AND (exp Hepatitis C/ OR hepatitis C.mp. OR exp hepatitis C antibody/ OR exp hepatitis C antigen/ OR exp Hepatitis C virus/ OR HCV.mp. OR hepacivirus.mp.)  **Systematic review of HCV in Pakistan [9]**  (esp. Pakistan/ OR Pakistan*.mp.) AND (exp Hepatitis C/ OR hepatitis C.mp. OR exp hepatitis C antibody/ OR exp hepatitis C antigen/ OR exp Hepatitis C virus/ OR HCV.mp. OR hepacivirus .mp.) |
| --- |

**References:**

1. Mohamoud YA, Mumtaz GR, Riome S, Miller D, Abu-Raddad LJ. The epidemiology of hepatitis C virus in Egypt: a systematic review and data synthesis. BMC infectious diseases. 2013;13(1):288.

2. Kouyoumjian SP, Chemaitelly H, Abu-Raddad LJ. Characterizing hepatitis C virus epidemiology in Egypt: systematic reviews, meta-analyses, and meta-regressions (under review). 2017.

3. Chemaitelly H, Chaabna K, Abu-Raddad LJ. The epidemiology of hepatitis C virus in the Fertile Crescent: systematic review and meta-analysis. PloS one. 2015;10(8):e0135281.

4. Chemaitelly H, Mahmud S, Rahmani AM, Abu-Raddad LJ. The epidemiology of hepatitis C virus in Afghanistan: Systematic review and meta-analysis. International Journal of Infectious Diseases 2015;40:54-63.

5. Fadlalla FA, Mohamoud YA, Mumtaz GR, Abu-Raddad LJ. The epidemiology of hepatitis C virus in the Maghreb region: systematic review and meta-analyses. PloS one. 2015;10(3):e0121873.

6. Mohamoud YA, Riome S, Abu-Raddad LJ. Epidemiology of hepatitis C virus in the Arabian Gulf countries: Systematic review and meta-analysis of prevalence. International Journal of Infectious Diseases. 2016;46:116-25.

7. Chaabna K, Kouyoumjian SP, Abu-Raddad LJ. Hepatitis C virus epidemiology in Djibouti, Somalia, Sudan, and Yemen: systematic review and meta-analysis. PloS one. 2016;11(2):e0149966.

8. Mahmud S, Akbarzadeh V, Abu-Raddad L. The epidemiology of hepatitis C virus in Iran: Systematic review and meta-analyses (under review). 2017.

9. Al-Kanaani Z MS, Abu-Raddad L. The epidemiology of hepatitis C virus in Pakistan: systematic review and meta-analyses (under preparation). 2017.
